# Factors associated with serious abdominal conditions in geriatric patients visiting the emergency department

**DOI:** 10.1186/s12873-024-00934-x

**Published:** 2024-01-25

**Authors:** Ar-aishah Dadeh, Wasitthee Uppakarnnuntakul

**Affiliations:** Department of Emergency Medicine, Faculty of Medicine, Songklanagarind Hospital, Prince of Songkla University, 90110 Hat Yai, Songkhla Thailand

**Keywords:** Serious abdominal conditions, Geriatric patients, Emergency department, Invasive procedure

## Abstract

**Background:**

Abdominal pain occurs in 20% of geriatric patients who visit the emergency department (ED). Geriatric patients usually have more severe conditions and a higher mortality rate. We aimed to determine the factors associated with serious abdominal conditions in geriatric patients who visit the ED with abdominal pain.

**Methods:**

This retrospective cohort study was conducted from January 1, 2017 to June 30, 2021. The inclusion criteria were patients aged ≥ 65 years and presented at the ED with acute abdominal pain. Significantly associated factors for serious abdominal conditions were examined using univariate and multivariate logistic regression analyses.

**Results:**

A total of 1221 patients were included in this study. Multivariate logistic regression analysis showed that the significant factors associated with serious abdominal conditions were male (adjusted odds ratio [AOR] 2.29, 95% CI:1.3–4.04; *p* = 0.004), anorexia (AOR 2.16, 95% CI:1.08–4.32; *p* = 0.03), NEWS 5–6 (AOR 2.96, 95% CI:1.35–6.49; *p* = 0.007), SBP 100–125 mmHg (AOR 1.5, 95% CI:0.75–2.99; *p* ≤ 0.001), guarding (AOR 6.92, 95% CI:3.39–14.12; *p* ≤ 0.001), WBC ≥ 14,000 cells/mm^3^ (AOR 2.08, 95% CI:1.06–4.09; *p* = 0.034), ED length of stay (EDLOS) 4–8 h (AOR 2.17, 95% CI:1.08–4.36; *p* = 0.03), and EDLOS ≥ 8 h (AOR 3.22, 95% CI:1.15–9; *p* = 0.025).

**Conclusions:**

The statistically significant factors associated with serious abdominal conditions in geriatric patients were male, anorexia, NEWS 5–6, SBP 100–125 mmHg, guarding, WBC ≥ 14,000 cells/mm^3^, EDLOS 4–8 h, and EDLOS ≥ 8 h.

**Supplementary Information:**

The online version contains supplementary material available at 10.1186/s12873-024-00934-x.

## Background

The elderly population has become a growing segment worldwide, which has brought about the so-called aging society. The elderly population was 927 million (9.1%) globally in 2019. The percentage of the elderly is expected to increase to 12% in 2030, 16% in 2050, and 23% in 2100 [1]. According to the National Statistical Office of Thailand, the percentage of elderly persons was 11.4% in 2020 [2]. Abdominal pain in geriatric patients accounts for 20% of geriatric patients who visit the emergency department (ED). Geriatric patients usually have more severe conditions that has resulted in a seven times higher mortality rate (11–14%), and 30% of geriatric patients received surgical treatment [3, 4]. Furthermore, if surgery is delayed the mortality and complication rates were reported to be 9% and 38.9%, respectively [5]. These manifestations can be explained by a lower immune response, several comorbidities, and a tortuous presentation. The number of ED visits increases with age and therefore 42% of geriatric patients visit one time per year and 8.2% of geriatric patients visit more than five times per year [6]. Variables associated with poor outcomes included age > 84 years, bandemia, intra-abdominal free air, hypotension, abnormal bowel sounds, dilated loops of bowel, and extreme leukocytosis [7]. Laboratory data used to diagnose acute appendicitis and bowel ischemia include red blood cell distribution width (RDW), mean platelet volume (MPV), and lactate but laboratory data cannot predict the outcome of serious abdominal conditions [8–11].

Moreover, the mortality rate following emergency surgery ranges from 15 to 30%, which doubles if comorbidities are present and can be significantly higher in patients who are over 75 years old [12].

To the best of our knowledge, factors that predict serious abdominal conditions have not been explored. This study aimed to determine the factors associated with serious abdominal conditions in geriatric patients who visit the ED with acute abdominal pain.

## Materials and methods

### Study design and setting

**Fig. 1:**
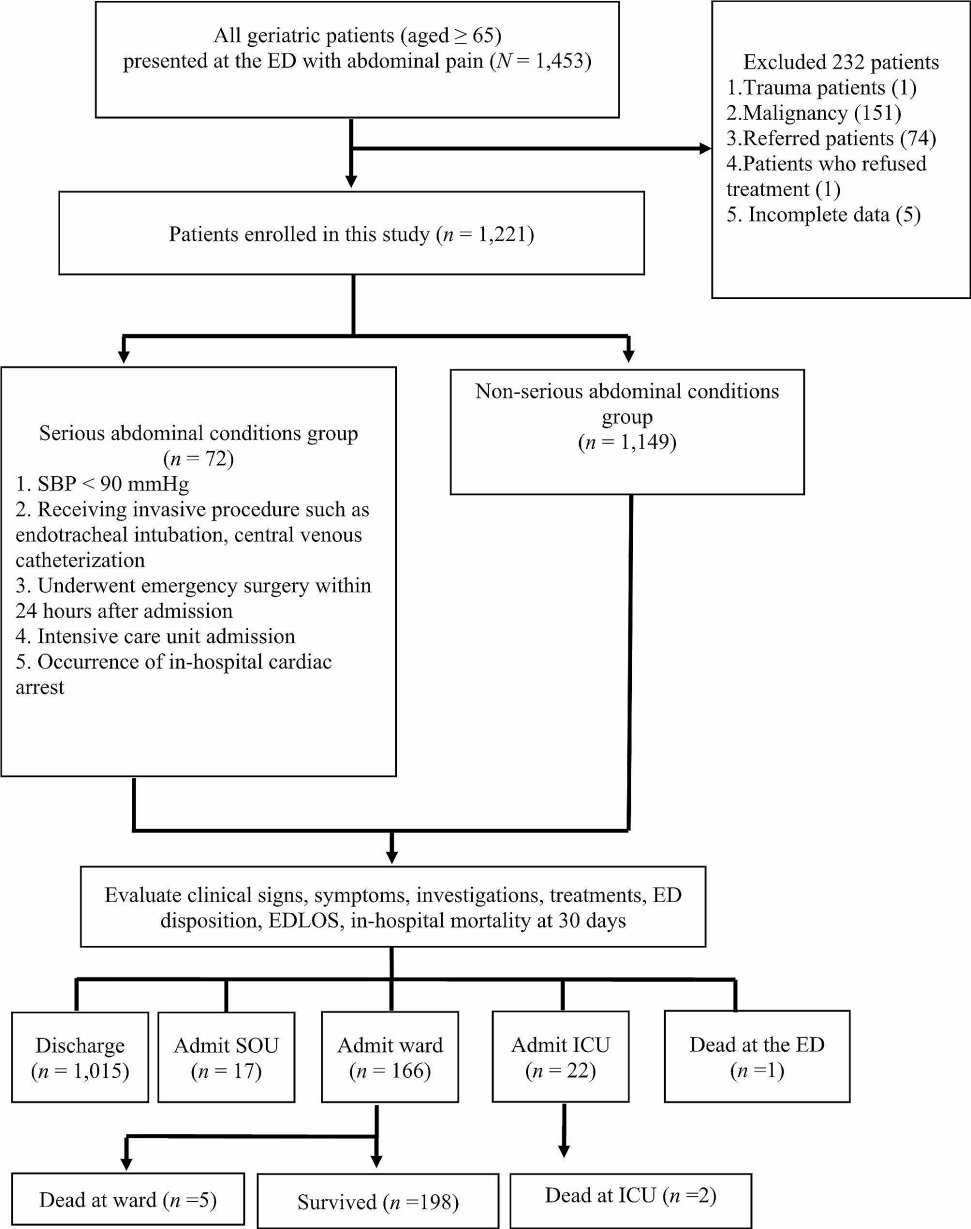
Study flow diagram of enrolled patients

This was a retrospective cohort study conducted in the ED of a tertiary care medical center with a capacity of 850 beds and is affiliated with a medical school. The data were collected from January 1, 2017 to June 30, 2021. The inclusion criteria were patients aged ≥ 65 years and presented at the ED with abdominal pain. The exclusion criteria were age < 65 years, trauma patients, patients with a malignant disease, referred patients, and patients who refused treatment. This study enrolled 1,221 elderly patients (Fig. 1).

### Data collection

The data collected from the electronic medical records and ED data records included baseline characteristics, onset of abdominal pain, associated symptoms, initial National Early Warning Score (NEWS), triage level, physical examination, laboratory investigations, diagnosis, treatment, ED length of stay (EDLOS), hospital length of stay (LOS), disposition, and 30-day in-hospital mortality. The patients were divided into two groups: serious abdominal conditions and non-serious abdominal conditions.

### Outcome measurement

The primary outcomes were factors associated with serious abdominal conditions in geriatric patients who came to the ED with abdominal pain. The secondary outcome was the mortality rate during admission.

### Statistical analysis

The n4Studies tool was used to determine the sample size of the study population to evaluate two independent proportions. The final calculated sample size was 410 patients. After adding a 10% dropout rate, the desired sample size was 451 patients. R software was used to perform the statistical analyses after all data were imported into EpiData. Continuous variables are reported as means and medians. Discrete variables are reported as percentages. The student’s t-test and Wilcoxon rank-sum test were used for continuous variables. Fisher’s exact test was used for discrete variables. After univariate logistic regression, a multivariate logistic regression model was used to evaluate factors associated with serious abdominal conditions. Significant factors (*p* < 0.1) were identified during univariate logistic regression. The associated factors were identified during multivariate logistic regression. The accuracy of factors was determined using the area under receiver operating characteristic curve (AUROC). Model discrimination was rated as unsatisfactory if the AUROC was between 0.5 and 0.6, satisfactory if the AUROC was between 0.6 and 0.7, good if the AUROC was between 0.7 and 0.8, very good if the AUROC was between 0.8 and 0.9, and excellent if the AUROC was between 0.9 and 1.0. Analytical results were described as odds ratio (OR) with 95% confidence interval (CI). Statistical significance was defined as *p*-value < 0.05.

### Operational definitions

A serious abdominal condition was defined as abdominal pain with at least one of the following: (1) a systolic blood pressure (SBP) < 90 mmHg; (2) invasive procedure such as endotracheal intubation and central venous catheterization; (3) emergency surgery; (4) intensive care unit (ICU) admission; and (5) cardiac arrest. The mortality rate was defined as the rate of patient death at 30 days after admission. Emergency surgery was defined as surgery that occurred within 24 h after admission.

### Compliance with ethical requirements

This study was approved by the Ethics Committee of Prince of Songkla University (approval number: REC 64-252-20-4). The Institutional Review Board of Prince of Songkla University is affiliated with the International Conference on Harmonization in Good Clinical Practice. The requirement for informed consent was waived in accordance with our institutional review board’s policy because the participants had no greater than minimum risk and the patients received standard medical care. All research information was kept confidential with limited data access by only the researcher and assistant. This study was conducted in accordance with the principles of Declaration of Helsinki.

## Results

### Patient characteristics and demographic data

A total of 1,453 geriatric patients with abdominal pain presented at the ED during the study period. Of these, 1,221 patients met the enrollment criteria. Seventy-two patients (5.9%) were categorized into the serious abdominal conditions group, and 1,149 patients (94.1%) were in the non-serious abdominal conditions group. The enrolled patients included 554 (45.4%) males and 667 (54.6%) females. The baseline characteristics of the serious abdominal conditions group and non-serious abdominal conditions group are shown in Table 1. The median age (interquartile range [IQR]) of the serious abdominal conditions group was younger than the non-serious abdominal conditions group (73 [69,80.2] vs. 74 [69,81]) without statistical significance. A comparison of the two groups revealed that the significantly different factors were sex, beta-blocker use, history of abdominal surgery, Emergency Severity Index (ESI) triage level, treatment given, EDLOS, ED disposition, hospital discharge status, and in-hospital mortality. The clinical findings, laboratory results, and complications of the geriatric patients who visited at the ED with abdominal pain are shown in Table 2. Significant presenting symptoms were fever, nausea or vomiting, hematemesis, and anorexia. The initial vital signs at the triage area that were found to be significant were SBP and respiratory rate (RR). Differences in the physical examination findings between the two groups were presence of tenderness point whether right lower quadrant (RLQ), left lower quadrant (LLQ), epigastrium, or suprapubic area, guarding, and abnormal bowel sounds. The median white blood cell (WBC) count in the serious abdominal conditions group was significantly higher than in the non-serious group (11,150 vs. 9,240 cells/mm^3^).

**Table 1:** Baseline characteristics geriatric patients who visited the ED with abdominal pain

Characteristics	Serious abdominal conditions (*n* = 72)	Non-serious abdominal conditions (*n* = 1,149)	Total (*n* = 1,221)	*p*-value
Sex				0.031
Male	42 (48)	512 (44.6)	554 (45.4)	
Female	30 (52)	637 (55.4)	667 (54.6)	
Age (years), median (IQR)	73 (69,80.2)	74 (69,81)	74 (69,81)	0.776
Age group				0.843
65–79	52 (72.2)	814 (70.8)	866 (70.9)	
80–89	18 (25)	287 (25)	305 (25)	
≥90	2 (2.8)	48 (4.2)	50 (4.1)	
Comorbidities				
DM	17 (23.6)	331 (28.8)	348 (28.5)	0.416
Hypertension	44 (61.1)	649 (56.5)	693 (56.8)	0.518
CKD	8 (11.1)	150 (13.1)	158 (12.9)	0.767
Cardiovascular disease	14 (19.4)	222 (19.3)	236 (19.3)	1.000
Cerebrovascular disease	10 (13.9)	125 (10.9)	135 (11.1)	0.551
Hepatobiliary disease	6 (8.3)	115 (10)	121 (9.9)	0.796
Asthma/COPD	8 (11.1)	74 (6.4)	82 (6.7)	0.196
Current medications				
Beta-blocker	22 (30.6)	226 (19.7)	248 (20.3)	0.038
Pain control	4 (5.6)	87 (7.6)	91 (7.5)	0.689
Immunosuppressant	1 (1.4)	28 (2.4)	29 (2.4)	0.867
Anticholinergic	1 (1.4)	21 (1.8)	22 (1.8)	1.000
Antihistamine	4 (5.6)	64 (5.6)	68 (5.6)	1.000
History of abdominal surgery				0.002
Present	22 (30.6)	182 (15.8)	204 (16.7)	
Absent	50 (69.4)	967 (84.2)	1017 (83.3)	
Onset of abdominal pain (hours) median (IQR)	15.5 (4.48)	24 (5,48)	24 (5,48)	0.825
ESI Triage levels				< 0.001
1	7 (9.7)	0 (0)	7 (0.6)	
2	21 (29.2)	137 (11.9)	158 (12.9)	
3	42 (58.3)	850 (74)	892 (73.1)	
4–5	2 (2.8)	162 (14.1)	162 (13.5)	
Treatment				< 0.001
Conservative	19 (26.4)	104 (9.1)	123 (10.1)	
Surgical	50 (69.4)	22 (1.9)	72 (5.9)	
Supportive	1 (1.4)	1023 (89)	1024 (83.9)	
Resuscitation	2 (2.8)	0 (0)	2 (0.2)	
EDLOS (hours), median (IQR)	6 (4,7.6)	3 (2,4.5)	3 (2,4.5)	< 0.001
< 4 h	14 (19.4)	753 (67)	767 (64.1)	
4–8 h	49 (68.1)	339(30.2)	388 (32.4)	
> 8 h	9 (12.5)	32 (2.8)	41 (3.4)	
Emergency department disposition				< 0.001
Admit ICU	22 (30.6)	0 (0)	22 (1.8)	
Admit ward	48 (66.7)	118 (10.3)	166 (13.6)	
Admit SOU	0 (0)	17 (1.5)	17 (1.4)	
Discharge	1 (1.4)	1014 (88.3)	1015 (83.1)	
Dead	1 (1.4)	0 (0)	1 (0.1)	
Hospital length of stay (days)	3 (97.2)	5.6 (11.7)	8.5 (16.8)	< 0.001
Hospital discharge status				< 0.001
Survived	63 (90)	135 (100)	198 (96.6)	
Dead	7 (10)	0 (0)	7 (3.4)	

Data are presented as *n* (%) unless otherwise indicated

*IQR* interquartile range, *DM* diabetes mellitus, *CKD* chronic kidney disease, *COPD* chronic obstructive pulmonary disease, *ESI* Emergency Severity Index, *EDLOS* emergency department length of stay, *ICU* intensive care unit, *SOU* short-stay observation unit

**Table 2:** Clinical findings, laboratory results and complications of geriatric patients who visited at the ED with abdominal pain

Characteristics	Serious abdominal conditions (*n* = 72)	Non-serious abdominal conditions (*n* = 1,149)	Total (*n* = 1,221)	*p*-value
Associated symptoms				
Fever	18 (25)	80 (7)	98 (8)	< 0.001
Nausea or vomiting	31 (43.1)	355 (30.9)	386 (31.6)	0.043
Hematemesis	2 (2.8)	1 (0.1)	3 (0.2)	0.001
Diarrhea	14 (19.4)	131 (11.4)	145 (11.9)	0.063
Hematochezia	0 (0)	5 (0.4)	5 (0.4)	1.000
Anorexia	18 (25)	122 (10.6)	140 (11.5)	< 0.001
Alteration of consciousness	1 (1.4)	4 (0.3)	5 (0.4)	0.696
NEWS, median (IQR)	3 (1.8,5)	2 (0,3)	2 (0,3)	< 0.001
NEWS group				< 0.001
0–4	50 (69.4)	1084 (94.3)	1134 (92.9)	
5–6	15 (20.8)	55 (4.8)	70 (5.7)	
≥7	7 (9.7)	10 (0.9)	17 (1.4)	
Initial vital signs				
Body temperature (^o^C), median (IQR)	36.6 (36.2,37.3)	36.6 (36.2,36.9)	36.6 (36.2,36.9)	0.166
SBP (mmHg), median (IQR)	146 (110,162.5)	149 (134,166)	149 (133,166)	0.045
HR (bpm), median (IQR)	78 (68,92)	76 (66,88)	76 (66,88)	0.269
RR (bpm), median (IQR)	24 (20,28)	22 (20,24)	22 (20,24)	< 0.001
SpO_2_ (%), median (IQR)	98 (96,100)	98 (97,100)	98 (97,100)	0.197
Abdominal examination findings				
Abdominal distension	22 (31)	236 (20.5)	258 (21.1)	0.052
Tender	59 (83.1)	806 (70.1)	865 (70.9)	0.028
Location of tenderness				
RUQ	10 (16.9)	128 (15.9)	138 (16)	0.974
LUQ	3 (5.1)	62 (7.7)	65 (7.5)	0.633
RLQ	28 (47.5)	129 (16)	157 (18.2)	< 0.001
LLQ	4 (6.8)	159 (19.7)	163 (18.8)	0.022
Epigastrium	7 (11.9)	218 (27)	225 (26)	0.016
Suprapubic	0 (0)	92 (11.4)	92 (10.6)	0.012
Umbilicus	7 (11.9)	59 (7.3)	66 (7.6)	0.31
Generalized	7 (11.9)	74 (9.2)	81 (9.4)	0.652
Bowel sounds				< 0.001
Normoactive	49(69)	923 (80.3)	972 (79.7)	
Hypoactive	10 (14.1)	42 (3.7)	52 (4.3)	
Hyperactive	12 (16.9)	184 (16)	196 (16.1)	
Guarding	19 (26.8)	38 (3.3)	57 (4.7)	< 0.001
Investigations				
CBC *n* = 611 (50)				
WBC (cells/µL), median (IQR)	11,150 (7730,14197.5)	9240 (7.45,11900)		0.005
PMN (%), median (IQR)	78.3 (69.3,86.6)	74 (62.3,82)		0.004
Bands (%), median (IQR)	0 (0,9)	0 (0,0)		< 0.001
MPV (fL), median (IQR)	9.9 (9.4,10.6)	9.9 (9.3,10.6)		0.812
RDW (%), median (IQR)	13.9 (13,15.2)	13.5 (12.8,14.6)		0.065
Lactate *n* = 153 (12.53%)				
Lactate (mmol/L), median (IQR)	1.7 (1.1,2.4)	1.2 (0.9,1.9)		0.008

Data are presented as *n* (%) unless otherwise indicated

*NEWS* National Early Warning Score, *IQR* interquartile range, *SpO*_*2*_ oxygen saturation, *RUQ* right upper quadrant, *LUQ* lower upper quadrant, *RLQ* right lower quadrant, *LLQ* left lower quadrant, *CBC* complete blood count, *WBC* white blood cell, *PMN* polymorphonuclear neutrophil, *MPV* mean platelet volume, *RDW* red blood cell distribution width

The three main diagnoses in the serious abdominal conditions group were (1) acute appendicitis (37.5%) that included 15 (20.8%) uncomplicated patients and 12 (16.7%) complicated patients, (2) abdominal aortic aneurysm (19.2%, 14 patients), and (3) acute cholecystitis (7%) that included 3 (4.2%) uncomplicated patients and 2 (2.8%) gangrenous patients (Table 3).

**Table 3:** Diagnoses of abdominal pain in geriatric patients who visited the emergency department

Characteristics	Serious abdominal conditions (*n* = 72)	Non-serious abdominal conditions (*n* = 1,149)	Total (*n* = 1,221)
Gastrointestinal conditions (medical)			
Dyspepsia/GERD	0 (0)	244 (21.2)	244 (20)
Constipation	0 (0)	111 (9.7)	111 (9.1)
Gastroenteritis/Infective diarrhea	0 (0)	132 (11.5)	132 (10.8)
Others	0 (0)	75 (6.5)	75 (6.2)
Gastrointestinal conditions (surgical)			
Volvulus	2 (2.8)	0 (0)	2 (0.2)
Intestinal obstruction	4 (5.6)	14 (1.2)	18 (1.5)
Uncomplicated diverticulitis	0 (0)	15 (1.3)	15 (1.2)
Ruptured diverticulitis	1 (1.4)	1 (0.1)	2 (0.2)
Acute uncomplicated appendicitis	15 (20.8)	0 (0)	15 (1.2)
Complicated appendicitis	12 (16.7)	3 (0.3)	15 (1.2)
Gastrointestinal bleeding	2 (2.8)	3 (0.3)	5 (0.5)
Spontaneous bacterial peritonitis	1 (1.4)	1 (0.1)	2 (0.2)
Hollow viscous perforation	3 (4.2)	1 (0.1)	4 (0.3)
Others	2 (2.8)	12 (1)	14 (1.2)
Hepatobiliary tract diseases			
Gallstone/CBD stone	0 (0)	50 (4.3)	50 (4.1)
Pancreatitis	1 (1.4)	12 (1.0)	13 (1.1)
Acute cholangitis	3 (4.2)	18 (1.6)	21 (1.7)
Acute cholecystitis	3 (4.2)	21 (1.8)	24 (2.0)
Gangrenous cholecystitis	2 (2.8)	1 (0.1)	3 (0.2)
Other	0 (0)	4 (0.4)	4 (0.4)
Urological conditions			
Urinary tract infection	1 (1.4)	88 (7.7)	89 (7.3)
Calculus	0 (0)	126 (11)	126 (10.3)
Other	1 (1.4)	31 (2.8)	34 (2.8)
Gynecological conditions			
Ovarian tumor	0 (0)	1 (0.1)	1 (0.1)
Prolapse uterus	0 (0)	1 (0.1)	1 (0.1)
Cardiovascular and Pulmonary diseases	4 (5.2)	5 (0.5)	7 (0.8)
Vascular diseases			
Symptomatic AAA	9 (12.5)	6 (0.5)	15 (1.2)
Ruptured AAA	3 (4.2)	1 (0.1)	4 (0.3)
Intramural hematoma	0 (0)	1 (0.1)	1 (0.1)
Aortic dissection	2 (2.8)	0 (0)	2 (0.2)
Non-specific abdominal pain	1 (1.4)	141 (12.3)	142 (11.6)
Other conditions	0 (0)	30 (2.7)	30 (2.4)

Data are presented as *n* (%)

*GERD* gastroesophageal reflux disease, *CBD* common bile duct, *CAPD* continuous ambulatory peritoneal dialysis, *STEMI* ST-elevation myocardial infarction, *NSTEMI* non ST-elevation myocardial infarction, *AAA* abdominal aortic aneurysm

### Factors associated with serious abdominal conditions in geriatric patients

The univariate logistic regression analysis showed that factors associated with the occurrence of serious abdominal conditions with high ORs were NEWS ≥ 7 (OR 15.18, 95% CI: 5.55–41.53), ESI level 1–2 (OR 16.55, 95% CI: 3.87–70.75), presence of abdominal guarding (OR 10.68, 95% CI: 5.76–19.8), and EDLOS ≥ 8 h (OR 15.13, 95% CI: 6.1–37.54) (Table 4). Furthermore, significant factors associated with serious abdominal conditions identified on multivariate logistic regression analysis were male (AOR 2.29, 95% CI:1.3–4.04), anorexia (AOR 2.16, 95% CI:1.08–4.32), NEWS 5–6 (AOR 2.96, 95% CI:1.35–6.49), SBP 100–125 mmHg (AOR 1.5, 95% CI:0.75–2.99; *p* ≤ 0.001), guarding (AOR 6.92, 95% CI:3.39–14.12; *p* ≤ 0.001), WBC ≥ 14,000 cells/mm^3^ (AOR 2.08, 95% CI:1.06–4.09), EDLOS 4–8 h (AOR 2.17, 95% CI:1.08–4.36), and EDLOS ≥ 8 h (AOR 3.22, 95% CI:1.15–9.0) (Table 5). In this study, EDLOS longer than 4 h had an AUROC of 0.738 with an 81% sensitivity and 67% specificity (Table 6). Characteristics of the patients in the serious abdominal outcomes group are shown in Table 7.

**Table 4:** Univariate logistic regression analysis of factors associated with serious abdominal conditions

Variables	Odds ratio	95% CI	*p*–value
Sex: male	1.74	1.07**–**2.82	0.024
Curren000t medication			
Beta–blocker use	1.8	1.07**–**3.03	0.028
History of abdominal surgery	2.34	1.38**–**3.96	0.002
Associated symptoms			
Fever	4.45	2.49**–**7.95	< 0.001
Nausea or vomiting	1.69	1.04**–**2.74	0.033
Hematemesis	32.8	2.94**–**366.13	0.005
Diarrhea	1.88	1.02**–**3.46	0.004
Anorexia	2.81	1.59**–**4.94	< 0.001
NEWS score			
5–6	5.91	3.13**–**11.18	< 0.001
≥ 7	15.18	5.55**–**41.53	< 0.001
ESI triage levels			
1–2	16.55	3.87**–**70.75	< 0.001
3	4	0.96**–**16.7	0.057
4–5	ref	ref	ref
Vital signs			
SBP 100–125 mmHg	2.28	1.28**–**4.07	0.005
RR ≥ 25/min	2.89	1.73**–**4.82	< 0.001
Abdominal signs			
Abdominal distention	1.74	1.03**–**2.93	0.039
Tenderness	2.09	1.11**–**3.94	0.022
RLQ tenderness	4.74	2.75**–**8.17	< 0.001
LLQ tenderness	3.38	1.21**–**9.46	0.02
Epigastrium tenderness	2.75	1.23**–**6.16	0.014
Bowel sounds			
Hypoactive	4.48	2.12**–**9.47	< 0.001
Hyperactive	1.23	0.64**–**2.36	0.535
Guarding	10.68	5.76**–**19.8	< 0.001
Investigations			
WBC 12,000–14,000 (cells/mm^3^)	1.48	0.71**–**3.08	0.301
WBC ≥ 14,000 (cells/mm^3^)	2.6	1.43**–**4.72	0.002
RDW ≥ 13 (%)	1.35	0.75**–**2.44	0.302
MPV ≤ 10.4	1.05	0.6**–**1.84	0.867
Lactate 2–4 (mmol/L)	2.45	0.99**–**6.03	0.051
Lactate ≥ 4 (mmol/L)	5.39	1.01**–**28.84	0.049
EDLOS (hours)			
4–8	7.77	4.23**–**14.27	< 0.001
≥ 8	15.13	6.1**–**37.54	< 0.001

*NEWS* National Early Warning Score, *ESI* Emergency Severity Index, *SBP* systolic blood pressure, *RR* respiratory rate, *RLQ* right lower quadrant, *LLQ* left lower quadrant, *WBC* white blood cell, *RDW* red blood cell distribution width, *MPV* mean platelet volume, *EDLOS* emergency department length of stay, *LOS* length of stay

**Table 5:** Multivariate logistic regression analysis of factors associated with serious abdominal conditions

Variables	Crude OR (95% CI)	AOR (95% CI)		*p*–value
Sex: male	1.82 (1.12**–**2.96)	2.29 (1.3**–**4.04)		0.004
History of abdominal surgery	2.22 (1.3**–**3.78)	1.68 (0.9**–**3.15)		0.106
Associated symptoms				
Anorexia	2.79 (1.58**–**4.92)	2.16 (1.08**–**4.32)		0.03
NEWS				
5–6	5.89 (3.11**–**11.16)	2.96 (1.35**–**6.49)		0.007
≥ 7	15.13 (5.52**–**41.43)	2.16 (0.52**–**8.91)		0.288
ESI triage levels				
1–2	14.09 (3.29**–**60.34)	3.36 (0.69**–**16.5)		0.135
3	3.56 (0.85**–**14.85)	1.66 (0.37**–**7.52)		0.511
Vital signs				
SBP < 100 mmHg	10.98 (3.9**–**30.94)	4.32 (0.96**–**19.36)		0.056
SBP 100–125 mmHg	2.33 (1.3**–**4.16)	1.5 (0.75**–**2.99)		< 0.001
Abdominal signs				
Guarding	10.44 (5.63**–**19.35)	6.92 (3.39**–**14.12)		< 0.001
Investigations				
WBC 12,000–14,000 (cells/mm^3^)	1.5 (0.72**–**3.14)	1.05 (0.44**–**2.47)		0.915
WBC ≥ 14,000 (cells/mm^3^)	2.64 (1.45**–**4.81)	2.08 (1.06**–**4.09)		0.034
EDLOS (hours)				
4–8	8.37 (4.48**–**15.64)	2.17 (1.08**–**4.36)		0.03
≥ 8	16.29 (6.49**–**40.9)	3.22 (1.15**–**9)		0.025

*OR* odds ratio, *CI* confidence internal, *AOR* adjusted odds ratio, *NEWS* National Early Warning Score, *ESI* Emergency Severity Index, *SBP* systolic blood pressure, *WBC* white blood cell, *EDLOS* emergency department length of stay

**Table 6:** Accuracy of characteristics, physical examination findings, and laboratory results associated with serious abdominal conditions in geriatric patients

Variables	AUROC	Sensitivity	Specificity	LR+ (95%CI)	LR− (95%CI)	PPV	NPV
Characteristics							
Male	0.5688642	0.58	0.55	1.31	0.75	0.08	0.96
Beta-blocker use	0.5544314	0.31	0.80	1.55	0.86	0.09	0.95
History of abdominal surgery	0.5735785	0.31	0.84	1.93	0.83	0.11	0.95
Fever	0.5901871	0.25	0.93	3.59	0.81	0.18	0.95
Nausea or vomiting	0.5607956	0.43	0.69	1.39	0.82	0.08	0.95
Hematemesis	0.5134537	0.03	1.00	31.92	0.97	0.67	0.94
Diarrhea	0.5402161	0.19	0.89	1.71	0.91	0.10	0.95
Anorexia	0.5719104	0.25	0.89	2.35	0.84	0.13	0.95
NEWS ≥ 5	0.6259126	0.31	0.94	5.40	0.74	0.25	0.96
ESI levels 1–2	0.6348274	0.39	0.88	3.26	0.69	0.17	0.96
Physical examinations							
SBP (mmHg)	0.5906054	0.32	0.86	2.27	0.79	0.12	0.95
RR ≥ 25 (breath/min)	0.596857	0.35	0.84	2.22	0.77	0.95	0.12
Abdominal distention	0.5522316	0.31	0.79	1.51	0.87	0.09	0.95
Abdominal tenderness	0.5647532	0.83	0.30	1.18	0.57	0.07	0.97
RLQ tenderness	0.6572633	0.47	0.84	2.97	0.63	0.18	0.96
LLQ tenderness	0.5647369	0.07	0.80	0.34	1.16	0.02	0.92
Epigastrium tenderness	0.5759137	0.12	0.73	0.44	1.21	0.03	0.92
Suprapubic tenderness	0.557072	0.00	0.89	0.00	1.13	0.00	0.92
Abnormal bowel sounds	0.5565832	0.31	0.80	1.58	0.86	0.09	0.95
Guarding	0.6172667	0.27	0.97	8.09	0.76	0.33	0.96
Laboratory results							
WBC ≥ 14,000 cells/mm^3^	0.567937	0.26	0.87	2.06	0.84	0.22	0.90
RDW ≥ 13 (%)	0.5285879	0.77	0.28	1.08	0.80	0.12	0.90
Lactate ≥ 2 mmol/L	0.6072184	0.42	0.80	2.05	0.73	0.34	0.84
EDLOS ≥ 4 h	0.7377422	0.81	0.67	2.44	0.29	0.14	0.98

*AUROC* area under receiver operating curve, *LR +* positive likelihood ratio, *LR −* negative likelihood ratio, *CI* confidence interval, *PPV* positive predictive value, *NPV* negative predictive value, *NEWS* National Early Warning Score, *ESI* Emergency Severity Index, *SBP* systolic blood pressure, *RR* respiratory rate, *RLQ* right lower quadrant, *LLQ* left lower quadrant, *WBC* white blood cell, *RDW* red blood cell distribution width, *EDLOS* emergency department length of stay, *LOS* length of stay

**Table 7:** Characteristics of the patients in the serious abdominal outcomes group

	Survived (*n* = 64)	Dead (*n* = 8)	Total (*n* = 72)
Shock (septic and hypovolemic)	13 (20.3)	6 (75)	19 (26.4)
Invasive procedure performed			
Central venous catheter insertion	5 (7.8%)	4 (50%)	9 (12.5%)
Mechanical ventilation	9 (14.1)	7 (87.5)	16 (22.2)
Emergency surgery	40 (62.5)	0 (0)	40 (55.6)
Exploratory laparotomy	6 (9.3)	1(12.5)	7 (9.2)
Appendectomy	26 (40.6)	0 (0)	40 (36.1)
Laparoscopy	4 (6.25)	0 (0)	4 (5.6)
Cholecystectomy	2 (3.1)	0 (0)	2 (2.8)
Other procedures	2 (3.1)	0 (0)	2 (2.8)
Intensive care unit admission	20 (31.3)	2 (25)	22 (30.6)
In-hospital cardiac arrest	0 (0)	8 (100)	8 (11.1)

Data are presented as *n* (%)

## Discussion

There is no clear definition for serious abdominal conditions. However, in our study we defined serious abdominal conditions as patients who had at least one of the following: SBP ≤ 90 mmHg; needed intubation or central line insertion or both; surgical procedure; ICU admission; or cardiac arrest. Several patients presented to the ED with a variety of primary complaints, including altered level of consciousness, fever, vomiting, and cardiac arrest, yet their diagnoses were intra-abdominal diseases. Early identification of these patients may decrease morbidity and mortality [4, 7]. The main results of the present study in multivariate logistic regression revealed that the statistically significant associated factors with serious abdominal conditions in geriatric patients were male, anorexia, NEWS 5–6, SBP 100–125 mmHg, presence of abdominal guarding, WBC ≥ 14,000 cells/mm^3^, and EDLOS 4–8 h and ≥ 8 h.

Male gender was explored upon multivariate analysis as one of associated factors with serious abdominal conditions with an AOR of 2.29. To our knowledge, there is no direct related study on the association between male gender and progressing to serious abdominal conditions in older adults. However, one previous study reported male gender was an independent risk factor associated with increased risk of major infections following trauma [13]. Alteration of hormonal function led to susceptibility to sepsis in older male adults [13]. Schröder et al., proposed that increased estradiol levels in both men and postmenopausal women were associated with sepsis. The source of estradiol in these patients was postulated to be from conversion of testosterone or decreased hepatic estrogen catabolism related to sepsis [14].

Obtaining a history from older adults has some limitations, such as hearing disorder, decreased vision, and impaired cognition, that may affect the ability to obtain an adequate clinical history [7]. We explored anorexia or loss of appetite as one of significant presenting symptoms in the serious abdominal conditions group. Our analysis showed that 37.5% of the serious abdominal conditions group were diagnosed with acute appendicitis. Acute abdominal pain with anorexia are common clinical indicators of acute appendicitis in all age groups. However, clinical indicators of acute appendicitis may not always be evident in elderly individuals; however, symptoms of peritonitis, such as abdominal distention, decreased abdominal wall movement, severe tenderness, and localized and generalized guarding, are more obvious [31]. Other essential data in history taking, which should be taken into account, that were identified on univariate analysis were fever (OR 4.45, *p* < 0.001), hematemesis (OR 32.8, *p* = 0.005), beta-blocker use (OR 1.8, *p* = 0.028), and history of abdominal surgery (OR 2.34, *p* = 0.002).

History of having fever is not a reliable marker for serious disease, and the elderly may be hypothermic in the presence of serious abdominal infections [7]. One study showed that 30% of patients over the age of 80 with intra-abdominal pathology that required surgery developed no fever [15]. On the other hand, a study by Potts et al. [16] showed that increased temperature was significant in cholecystitis and perforation. The present study showed two common diagnoses in the serious abdominal conditions group: acute appendicitis (37.5%) and acute cholecystitis (11.2%). We assume that the presence of fever in elderly patients may indicate a serious abdominal pathology requiring surgery.

Acute abdominal pain and hematemesis are indications of upper gastrointestinal bleeding in patients who present at the ED. Our results showed that hematemesis had the highest OR (OR 32.8, *p* = 0.005) on univariate analysis, which was associated with serious abdominal conditions. One retrospective observational study showed that the most common cause of gastrointestinal bleeding in the elderly was peptic ulcer, which had a 28-day mortality rate of 14%. They also demonstrated that the most important predictor of in-hospital mortality for geriatric patients with gastrointestinal bleeding was hemodynamic instability at the time of ED presentation [17].

Beta-blockers are commonly used for several medical conditions such as hypertension, arrhythmia, migraine, glaucoma, and anxiety [18]. Moreover, beta-blockers are also prescribed as the primary prophylactic agent for upper gastrointestinal bleeding in cirrhotic patients from variceal bleeding [19]. Most patients in our study population had hypertension (693 patients, 56.8%) and cardiovascular diseases (236 patients, 19.3%). Of these, 248 (20.3%) patients were taking beta-blockers. These patients may not have a tachycardic response to hypovolemia, which may lead to a delay in the diagnosis and treatment of shock [20].

To date, few studies have demonstrated an association between a history of previous abdominal surgery and serious abdominal conditions. One retrospective study concluded that previous abdominal surgery in elderly patients with colorectal cancer may lead to a prolonged laparoscopic procedure and prolonged exposure to anesthetic agents, but no evidence of increased hospital mortality or morbidity [21]. Comorbidities were reported as a predictor of increased hospital mortality and adverse events in geriatric patients [3, 4]. However, our study showed no significant difference of comorbidities between the two groups.

Defining shock in elderly patients is different from young adults. Using the criteria of SBP < 90 mmHg may be inadequate and may delay a diagnosis. The present study showed that SBP of 100–125 mmHg was significantly associated with serious abdominal conditions (AOR 1.5, *p* < 0.001). Our findings are in concordance with one large retrospective study (*n* = 902,852 patients), which aimed to determine the blood pressure which was best associated with worse outcomes and mortality in adult trauma patients. They concluded that in patients younger than 65 years, the classic definition of hypotension as an ED SBP < 90 mm Hg remains optimal. However, in patients older than 65 years, an SBP threshold of 117 mmHg was identified as the more appropriate value to define hypotension in trauma patients [22]. On abdominal examination, our analysis found that abdominal guarding was associated with serious abdominal conditions that was more than six times greater than a healthy individual. However, values from a physical examination in elderly individuals may be lower due to physiologic changes brought on by aging. Abdominal wall muscle atrophy reduces rebound tenderness and abdominal guarding [15]. For healthcare providers involved in caring for geriatric patients, the presence of guarding should increase awareness of serious underlying abdominal conditions. Other abdominal findings associated with serious abdominal conditions were RLQ tenderness (OR 4.74, *p* < 0.001), LLQ tenderness (OR 3.38, *p* = 0.02), epigastrium tenderness (OR 2.75, *p* = 0.014), and hypoactive bowel sounds (OR 4.48, *p* < 0.001). Recognizing these physical signs increases the value of an early diagnosis and can lead to early definitive treatment in the elderly population.

A complete blood count is a common diagnostic tool in defining infection and the cause of abdominal pain in all age groups [4, 7]. Previous studies showed that geriatric patients failed to demonstrate leukocytosis in the state of infection due to the decline in the immune function against infection [5, 15]. The present study revealed that leukocytosis, defined as a WBC count ≥ 14,000 cells/mm^3^, doubled the AOR of being associated with serious abdominal conditions. A study by Asadollahi et al. reported that leukocytosis had a positive relationship with mortality in general hospitalized patients in all age groups [23]. MPV and RDW were reported to be potential parameters for the diagnosis of acute and perforated appendicitis [9]. A study by Fan et al. showed that the MPV value was reduced in acute gangrenous appendicitis [24]. In our study, both MPV and RDW were not significant factors for serious abdominal conditions. Further studies on the potential of MPV and RDW in detecting serious underlying abdominal conditions should be considered.

Several studies reported lactate as a useful biomarker in detecting surgical emergency in patients with acute abdominal disorders [11, 25, 26]. A lactate level ≥ 4 mmol/L was identified in univariate analysis with an OR of 5.39, which was associated with serious abdominal conditions with statistical significance but was no longer significant in multivariate analysis. However, lactate ≥ 2 mmol/L has only 42% sensitivity and 80% specificity in recognizing geriatric patients with serious abdominal conditions. The reason could be from the smaller number of lactate tests performed during the early years of this current study. However, the lactate test was widely performed in the later years of this study.

Among the ward and ED patients, NEWS is a well-validated measure to predict unexpected ICU admission, cardiac arrest, and mortality within 24 h [27]. NEWS is used for the early evaluation of infection and sepsis. Our study showed that a NEWS of 5–6 and higher corresponded to serious abdominal conditions. This level of NEWS correlated with ESI 1–2 triage levels. An ESI level 1–2 was identified on univariate analysis with an OR of 16.55 (*p* < 0.001) and was associated with the occurrence of serious abdominal conditions. Patients with abdominal pain who needed initial stabilization with lifesaving procedures for airway, breathing, and circulation may indicate a serious diagnosis that requires a surgical procedure [28].

Overcrowding in the ED reduces the ability to appropriately manage and treat critically ill patients. EDLOS is a critical statistic to assess the efficiency of ED management, and it is also a critical indicator of the efficiency of ED management [29]. No previous study has reported an association between EDLOS and serious abdominal conditions. However, more serious conditions might require more time for extensive investigations and treatment resulting in a longer ED stay. The mortality rate of geriatric patients who present with acute abdominal pain ranged from 11 to 14%. The reasons for the high mortality rate in geriatric patients were related to comorbidities, former surgical procedures, multiple drug use, impotent immune system, and delayed recognition of serious conditions in the ED [3]. A study by Özkan et al. reported a mortality rate of 14% in emergency abdominal surgery in geriatric patients. Our study reported a mortality rate of 0.7% but our results showed the mortality rate increased to 11.1% if one of serious abdominal conditions criteria presented. The difference in mortality rate could be due to different study groups. Our study involved all patients with medical and surgical conditions, and our study had a larger number of subjects than the referenced study, which involved only patients with abdominal surgery in a total of 92 patients [30]. The present study had a lower rate of performing surgical procedures compared to a previous study (5.9% vs. 17.6%) [3]. That study reported that malignancy related conditions were the leading causes of surgery (8%), and the final diagnoses related to abdominal pain were due to malignancy (9.8%). Our study found that appendectomy accounted for 52% of all surgical procedures; however, our study excluded malignancy related abdominal pain and 13% (4 patients) of acute appendicitis patients were treated conservatively. Conservative therapy was shown to be non-inferior to appendectomy in a recent large randomized study that compared antibiotics with appendectomy, which included patients with appendicolith [31].

### Limitations

There are several limitations of this study, First, it was retrospective in nature and conducted in a single center. Second, we did not perform a subgroup analysis of patients who presented with serious abdominal conditions and underwent emergency surgery, which may have revealed more specific information.

## Conclusions

The study revealed that the factors associated with serious abdominal conditions in geriatric patients were male, anorexia, NEWS 5–6, SBP 100–125 mmHg, guarding, leukocytosis (WBC ≥ 14,000 cells/mm^3^), EDLOS 4–8 h, and EDLOS ≥ 8 h.

### Electronic supplementary material

Below is the link to the electronic supplementary material.

**Additional file 1: Supplementary Table 1**. Characteristics of the patients in serious abdominal outcomes group

## Data Availability

The retrospective data used to support the findings of this study are available from the corresponding author upon request.

## Abbreviations

AAA: Abdominal aortic aneurysm
AOR: Adjusted odd ratios
AUROC: Area under receiver operating curve
CAPD: Continuous ambulatory peritoneal dialysis
CBD: Common bile duct
CI: Confidence interval
CKD: Chronic kidney disease
COPD: Chronic obstructive pulmonary disease
DM: Diabetes mellitus
ED: Emergency department
EDLOS: Emergency department length of stay
EGPA: Eosinophilic granulomatosis with polyangiitis
ESI: Emergency Severity Index
GERD: Gastroesophageal reflux disease
ICU: Intensive care unit
IQR: Interquartile range
LLQ: Left lower quadrant
LR-: Negative likelihood ratio
LR+: Positive likelihood ratio
LUQ: Left upper quadrant
MPV: Mean platelet volume
NEWS: National Early Warning Score
NPV: Negative predictive value
NSTEMI: Non ST-elevation myocardial infarction
OR: Odd ratios
PMN: Polymorphonuclear
PPV: Positive predictive value
PR: Pulse rate
RDW: Red blood cell distribution width
RLQ: Right lower quadrant
RUQ: Right upper quadrant
RR: Respiratory rate
SBP: Systolic blood pressure
SOU: Short-stay observation unit
SpO_2_: Oxygen saturation
STEMI: ST-elevation myocardial infarction
WBC: White blood cell
