# Supplementary material for: Factors associated with serious abdominal conditions in geriatric patients visiting the emergency department
Source: BMC Emerg Med. 2024 Jan 25;24:16. doi: 10.1186/s12873-024-00934-x (PMC10809639; doi:10.1186/s12873-024-00934-x)
Supplement: Supplementary file 1 — Additional file 1: Supplementary Table 1. Characteristics of the patients in serious abdominal outcomes group [file 12873_2024_934_MOESM1_ESM.docx]

**Additional file 1: Supplementary Table 1.** Characteristics of the patients in serious abdominal outcomes group

|  | **Survived**  **(*n* = 64)** | **Dead**  **(*n* = 8)** | **Total**  **(*n* = 72)** |
| --- | --- | --- | --- |
| Shock (septic and hypovolemic) | 13 (20.3) | 6 (75) | 19 (26.4) |
| Invasive procedure performed |  |  |  |
| • Central venous catheter insertion | 5 (7.8%) | 4 (50%) | 9 (12.5%) |
| • Mechanical ventilation | 9 (14.1) | 7 (87.5) | 16 (22.2) |
| Emergency surgery | 40 (62.5) | 0 (0) | 40 (55.6) |
| • Exploratory laparotomy | 6 (9.3) | 1(12.5) | 7 (9.2) |
| • Appendectomy | 26 (40.6) | 0 (0) | 40 (36.1) |
| • Laparoscopy | 4 (6.25) | 0 (0) | 4 (5.6) |
| • Cholecystectomy | 2 (3.1) | 0 (0) | 2 (2.8) |
| • Other procedures | 2 (3.1) | 0 (0) | 2 (2.8) |
| Intensive care unit admission | 20 (31.3) | 2 (25) | 22 (30.6) |
| In-hospital cardiac arrest | 0 (0) | 8 (100) | 8 (11.1) |

Data are presented as *n* (%).
